# Socio-demographic disparity in oral health among the poor: a cross sectional study of early adolescents in Kilwa district, Tanzania

**DOI:** 10.1186/1472-6831-10-7

**Published:** 2010-04-20

**Authors:** Kijakazi O Mashoto, Anne N Astrom, Marit S Skeie, Joyce R Masalu

**Affiliations:** 1 Institute of Clinical Odontology, University of Bergen, Bergen, Norway; 2 Centre for International Health, University of Bergen, Bergen, Norway; 3 National Institute for Medical Research, Tanzania; 4 Muhimbili University of Health Alliances and Science, Dar es Salaam, Tanzania

## Abstract

**Background:**

There is a lack of studies considering social disparity in oral health emanating from adolescents in low-income countries. This study aimed to assess socio-demographic disparities in clinical- and self reported oral health status and a number of oral health behaviors. The extent to which oral health related behaviors might account for socio-demographic disparities in oral health status was also examined.

**Methods:**

A cross-sectional study was conducted in Kilwa district in 2008. One thousand seven hundred and forty five schoolchildren completed an interview and a full mouth clinical examination. Caries experience was recorded using WHO criteria, whilst type of treatment need was categorized using the ART approach.

**Results:**

The majority of students were caries free (79.8%) and presented with a low need for dental treatment (89.3%). Compared to their counterparts in opposite groups, rural residents and those from less poor households presented more frequently with caries experience (DMT>0), high need for dental treatment and poor oral hygiene behavior, but were less likely to report poor oral health status. Stepwise logistic regressions revealed that social and behavioral variables varied systematically with caries experience, high need for dental treatment and poor self reported oral health. Socio-demographic disparities in oral health outcomes persisted after adjusting for oral health behaviors.

**Conclusions:**

Socio-demographic disparities in oral health outcomes and oral health behaviors do exist. Socio-demographic disparities in oral health outcomes were marginally accounted for by oral health behaviors. Developing policies and programs targeting both social and individual determinants of oral health should be an urgent public health strategy in Tanzania.

## Background

Social disparities in health- and oral health outcomes as measured by education, occupation, income and household assets or by indices derived by combining indicators constitute one of the main challenges for public health [1,2]. Contemporary evidence suggest that the lower the material standard of living, the worse the health status irrespective of the measure (being it clinically assessed or self reported) used to assess it [1-4]. Compared to adult populations, social disparities in health- and oral health among children and adolescents have received relatively little attention [5]. Torsheim *et al*. [4] observed substantial inequalities in adolescents' self-reported health status related to the distribution of family material resources across European- and North American countries. A pattern of poorer health was demonstrated among populations for whom family material resources were distributed less evenly. Recognizing oral health to be an integral part of general health, recent studies suggest a social gradient in oral health, with the magnitude of inequality being larger in some countries than in others [3,6,7]. The WHO International Collaborative studies (ICS-I or II), have demonstrated a social gradient in adolescents' caries experience across high-and low income countries and various oral health care systems [8]. Social- and behavioral inequality in dental caries and periodontal diseases has also been identified in comparative studies from sub Saharan Africa, South East Asian countries and Chile [8,9]. Moreover, social disparities in adolescents' oral health behavior have been demonstrated in developing countries and elsewhere, with oral health detrimental behaviors being most common in subjects of lower socio-demographic status [10-12]. Yet, mixed evidence exists with some studies reporting social disparity in adolescents' caries experience whereas others do not [5].

It is assumed that inequality has multiple causes and that the effect on oral health of socio-economic and demographic factors is mediated through environmental exposure, psychosocial factors, lifestyle and availability of health care services [13]. Petersen [8] presented a risk factor model for dental caries, suggesting that socio-environmental factors influence behavioral- and attitudinal factors which in turn impact on clinical- and subjective oral health outcomes. Socio-demographic factors, such as place of residence, age, gender, family income and education and individual factors in terms of oral health behaviors might influence oral health outcomes. Moreover, socio-demographic factors might influence oral health outcomes directly or indirectly through oral health related behaviors. Evaluating the mediating role of oral health behavior among adults in Australia, Sanders *et al*, [14] found that the slope of the socio-economic gradient in the Oral Health Impact Profile (OHIP 14) scores was significantly attenuated by dental visiting. Analyzing data from the 1998 Adult Dental Health Survey in the UK, Donaldson *et al *[15] concluded that the socio-economic gradient in the number of sound teeth was partially explained by dental attendance patterns. Sabbah *et al *[3] analyzed data from the US Third National Health and Nutrition Examination Survey focusing adults above 17 yrs and found attenuation of socio-economic disparities in oral health status after adjusting for various oral health related behaviors. Recent studies among US adolescents revealed that socio-economic disparities in caries experience could not be accounted for by similar disparities in oral health behaviors [5].

Tanzania has one of the poorest overall health indicators in the world [16]. Few studies have examined socio-economic disparities in oral health among adolescents in Tanzania where exposure to topical fluorides is inappropriate and where access to oral health care services at the local community level is at best very limited [17,18]. Improved understanding of this issue might have important policy- and oral health program implications in Tanzania where the oral health policy gives priority to children and adolescents as target groups for health care services. A previous study focusing the same study group as the present one revealed that substantial proportions of Tanzanian adolescents admitted untreated dental caries, reduced oral health related quality of life and treatment needs [19]. This study takes the analysis a step further by exploring the socio-demographic distribution of oral impairment, oral impacts and treatment needs among adolescents in Tanzania.

Focusing early adolescents attending primary school in Kilwa, south -eastern Tanzania, this study aimed to assess socio-demographic disparities in caries experience, treatment need, self-reported oral health status and a number of oral health related behaviors. The extent to which oral health related behaviors accounted for socio-demographic disparities in oral health status was also investigated.

## Method

### Study area

The present paper is based on data generated from a cross -sectional study carried out in the coastal region of Lindi in 2008 [19]. Lindi is one of the most sparsely populated regions of Tanzania main land with a population density of 66,046 per square km. The population was 791,306 as of the 2002 national census [20]. Lindi is divided into six districts; of which Kilwa (N = 171,850) was purposively selected for this study, since the fluoride concentration in water (0.2 mg/L) is low and since the district is particularly deprived regarding access to oral health care services. The entire Kilwa population is served by one assistant dental officer (1:171,850).

### Study population

The study population comprised of adolescents attending standard 6 in public primary schools (N = 8609) in Kilwa district. As this study included several outcomes, the size of the sample was calculated separately for each of them and the largest sample size required was adopted. A sample size of 2000 students was calculated to be satisfactory; assuming that the percentage of adolescents expected to have dental caries was 30%, using an absolute precision (d) of 0.03, 95% CI and a design factor of 2 [21]. A stratified disproportionate one stage cluster sample design with public primary schools as the primary sampling units were utilized. Kilwa district is divided into 18 rural (N = 7444 standard 6 pupils) and 2 urban (N = 1165 standard 6 pupils) wards. To reach the estimated sample size, 8 rural wards (8/18 = 0.4) were selected at the first stage by systematic random sampling. In addition, both urban wards were included in the sample. At the second stage, all standard 6 adolescents that were accessible in public primary schools in the selected wards were included in the sample. Twenty seven schools (17 rural schools, n = 1408 and 10 urban schools n = 1059) out of a total of 101 schools (N = 8609 standard 6 primary school subjects, rural = 7444 and urban = 1165) present in Kilwa district were invited to participate in the study (n = 2467). One thousand seven hundreds and eighty (1780/2465, response rate 72.6%) adolescents (mean age 13.8 yr, [sd 1.67]) consented to participate in the study. Being out of school at the time of data collection was the main reason for non-participation. Twelve adolescents below the age of 10-and above the age of 19 yr were excluded from the analysis. Moreover, 23 adolescents refused to be examined clinically because of fear of the dental instruments. A total of 837 urban (52.3% girls, mean age 13.4 [sd 1.62]) and 908 rural adolescents (48.5% girls, mean age 14.2 [sd 1.64]) completed an extensive personal interview and under-went a full mouth clinical examination.

Permission for adolescent's participation was sought from school authorities and parents. Ministry of Education and Vocational Training through the District Council approved the conduct of the study. Ethical clearance was granted by the National Institute for Medical Research in Tanzania and the Regional Committee for Medical Research Ethics and the Norwegian Data Inspectorate. Written and verbal informed consent was obtained from adolescents and their parents prior to study participation.

### Interview

A structured interview schedule was administered by trained research assistants and completed by students in face to face interviews. The interview schedule was originally constructed in English, translated to Kiswahili, the national language of Tanzania, and then back translated into English and pilot tested prior to its use in the field. Each interview was conducted in a private and quiet place outside the classroom.

*Self-reported oral health *was assessed by six items; "What do you think about the state of your teeth and mouth?" The responses ranged from (1) very good to (4) very bad. "How satisfied or dissatisfied are you with your teeth-, tooth appearance-, tooth colour-, position of teeth-, and chewing ability-"? The responses for the five questions regarding satisfaction ranged from (1) very satisfied to (4) very dissatisfied. A sum score was obtained by adding the six items. Subsequently this sum score was dichotomised on a median split into (1) good perceived oral health and (0) poor perceived oral health. *Parents' level of education *was originally scored from (1) no education to (6) college or university education. For analysis the variables (mother's and father's education) were recoded into (0) low education (including original categories 1 and 2) and (1) high education (including original categories 3, 4, 5 and 6). *Family wealth *was assessed as an indicator of socio-economic status according to a standard approach in equity analysis [22]. Durable household assets indicative of family wealth (i.e. bicycle, motorcycle, car, TV) were recorded as (1) "available and in working condition" or (0) "not available and/or not in working condition." These assets were analyzed using principal components analysis, PCA. The first component resulting from this analysis was used to categorize households into four approximate quartiles of wealth ranging from the 1^st ^poorest quartile to the least poor 4^th ^quartile. *Dental services utilization *was measured by the response to the question **"**Have you ever attended a dentist/dental therapist for treatment? The response was either yes (1) or no (0). *Frequency of sugar intake *was made up by a sum score of items assessing intake of (1) biscuits, (2) chocolates/toffee/sweets, (3) ice cream, (4) soda, (5) sugared tea/coffee and (6) sugared fruit juice. Each item was assessed on a scale ranging from (1) seldom or never to (5) more than once a day. A total score for sugar intake was computed by summing up the six items and subsequently dichotomized based on a median split into (0) low sugar intake and (1) high sugar intake. *Frequency of tooth brushing *was originally scored from (1) seldom or never to (5) more than once a day, and grouped into (0) never or seldom, (1) once a day and (2) more than once a day. *Use of toothpaste *was scored as (1) Yes and (0) No.

### Clinical assessment

To avoid inter examiner inconsistencies clinical examination was carried out by one trained and calibrated dentist (KOM). Each examination lasted for approximately 15 minutes and about 25-30 school pupils were examined each school day. Caries experience was assessed under field conditions with the examiner sitting on the school desk and adolescent lying on the desk with head resting on the examiner's laps. Dental probe and mouth mirror were used to detect caries. Cotton rolls were used to control saliva. Natural light was used as a source of illumination. Caries was scored using World Health Organization criteria [23]. A tooth was classified as carious if there was visual evidence of undermined enamel or cavity on either occlusal, proximal or smooth surface or both surfaces. A tooth was considered missing if there was a history of extraction because of pain and or a cavity prior to extraction. The sound category was used when there was no evidence on any surface of treated or untreated caries. For each student DMT was computed as the sum of decayed and missing permanent teeth, (no permanent tooth had filling). Examined adolescents were categorized into those who were caries free DMT = 0 and those with caries experience DMT>0. An overall judgement was made as to the kind of treatment each participant needed according to the ART approach [24]. Participants were divided into four groups of treatment need: (0) did not need treatment for caries, (1) requiring ART only (those diagnosed with single surface carious lesions with no history of pain and not tender to percussion), (2) requiring extraction (those with grossly damaged crown, pain when sleeping and tender to percussion) and (3) needing both extraction and ART. For the present analysis, two categories were formed in terms of (0) "no or low treatment need" (including the original categories 0 &1), and (1) "moderate to high treatment need" (including the original categories 2 & 3).

### Statistical analysis

Data were analysed using the Statistical Package for Social Science (Version 15.0.1). Cluster effect was adjusted for using STATA 10.0. Cross tabulations were tested by Chi-square statistics. Multiple variable analyses with caries experience,, treatment need and self-reported oral health as dependant variables were conducted using stepwise multiple logistic regression analyses and 95% Confidence intervals (CI). To examine whether oral health related behaviours accounted for socio-demographic disparities in clinical- and self reported oral health outcomes, the approach suggested by Baron and Kenny was adopted [25]. Thus, step II of the regression model adjusting for socio-economic measures, age, gender and oral health behaviours were compared to those of step I adjusting for socio-economic variables, age and gender, following assessment of the socio-demographic distribution of oral health and oral health behaviours separately. This method accounts for the direct and indirect effect of socio-demographic variables on oral health outcomes. Reduction in ORs for the socio-demographic variables from step I to step II was interpreted as evidence of mediation of effects, given that socio-economic characteristics varied systematically with oral health outcomes and oral health behaviours and that the relationship between oral health outcomes and oral health behaviours were statistically significant [25].

## Results

### Sample profile

**Figure 1:**
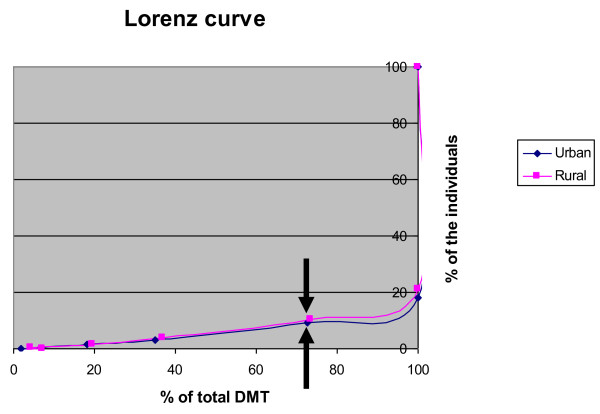
**Lorenz curve for caries distribution of early adolescents in urban and rural districts**. Each point of the curves denotes the proportion of the population (y-axis) responsible for the proportion of the total burden of caries lesions (x-axis).

The mean DMT scores were 0.37 (sd 0.85) and 0.32 (sd 0.79) in urban and rural adolescents, respectively. A total of 20.2% had DMT>0 and 1.4% had MT due to caries. Table 1 gives percentage distribution of participants' socio-demographics and the crude and weighted prevalence estimates of oral health behaviors, DMT, self reported oral health and treatment need. Significant caries index (SiC), which give the mean DMT of one third of the most severely affected group was 1.03 (not shown in the table). The Lorenz curve shown in Fig. 1, is a tool used to reflect if there are disparities in caries distribution between populations. In this case only small disparities in caries distribution were found between rural and urban population groups. Seventy five percent of the burden of caries was contributed by about 12% of individuals with a high level of disease in rural area and 10% of individuals with a high level of disease in urban area.

**Table 1:** Frequency distribution of adolescents' socio-economic characteristics and frequency distribution and weighted estimates of oral health behaviors and oral health outcomes.

	% (n)	Weighted estimates (%)
*Wealth index:*		
1^st ^quartile (Poorest)	26.4 (461)	
2^nd ^quartile	44.7 (780)	
3^rd ^quartile	4.0 (69)	
4^th ^quartile (Least poor)	24.9 (435)	
*Mother's education:*		
Low	44.7 (780)	
High	55.3 (965)	
*Father's education:*		
Low	41.6 (726)	
High	58.4 (1019)	
*Sex:*		
Boys	49.7 (867)	
Girls	50.3 (878)	
*Age:*		
10 - 14 years	67.9 (1184)	
15 - 19 years	32.1 (561)	
*Residence:*		
Urban	48.0 (837)	
Rural	52.0 (908)	
Oral health behaviors		
*Sugar consumption:*		
Low intake	45.7 (798)	55.0
High intake	54.3 (947)	45.0
*Tooth brushing frequency:*		
Never or seldom	21.9 (382)	24.9
Once a day	45.2 (789)	44.4
More than once a day	32.9 (574)	30.7
*Dental attendance:*		
Yes	10.4 (182)	8.3
No	89.6 (1563)	91.7
*Use of fluoridated toothpaste:*		
Yes	67.3 (1175)	63.7
No	32.7 (570)	36.3
*Caries experience*		
DMT > 0	20.2 (343)	20.9
DMT = 0	79.8 (1402)	79.1
*Treatment need*		
No/low	89.3 (1558)	88.6
Moderate/high	10.7 (187)	11.4
*Self-reported oral health:*		
Poor	42.8 (746)	43.1
Good	57.2 (999)	56.9

### Test-retest reliability

Duplicate clinical examinations were carried out on a randomly selected sub-sample of 20 participants. Re-examination took place after two weeks. The mean age of this subsample was 13.5 years (SD = 1.39). Analysis performed on the duplicate examination recordings gave weighted kappa statistics of 1.00 for missing teeth due to caries and decayed teeth.

### Socio-demographic distribution of oral health behaviors

Adolescents with highly educated mothers, younger subjects and urban residents presented with high sugar intake more frequently than their counterparts in the opposite groups (Table 2). Adolescents in the poorest wealth category had less often high sugar intake and were more frequently no users of tooth brushing and fluoridated toothpaste compared with those in the least poor wealth category. After controlling for variation in wealth index, parental education, age, gender and place of residence in multiple variable logistic regression analysis, the following independents maintained statistical significance; wealth index, parental education and place of residence with respect to sugar intake, wealth index and place of residence with respect to tooth brushing, gender and place of residence with respect to dental attendance and wealth index, gender and place of residence with respect to use of fluoridated toothpaste (not shown in table).

**Table 2:** Distribution of oral health behaviors by socio-demographic characteristics.

Variable	High sugar intake	Never/seldom tooth brushing	Dental attendance	No fluoridate toothpaste
	**% (n)**	**% (n)**	**% (n)**	**% (n)**
*Wealth index:*				
1^st ^poorest quartile	48.6 (224)	26.5 (122)*	8.2 (38)	38.0 (175)**
2^nd ^poor quartile	47.7 (372)	22.3 (174)	8.8 (69)	35.6 (278)
3^rd ^poor quartile	59.4 (41	20.3 (14)	21.7 (15)	33.3 (23)
4^th ^least poor quartile	71.3 (310)**	16.6 (72)	13.8 (60)**	21.6 (94)
*Mother's education:*				
Low	49.4 (385)	24.2 (189)	10.0 (78)	35.9 (280)*
High	58.2 (562)**	20.0 (193)	10.8 (104)	30.1 (290)
*Father's education:*				
Low	52.2 (379)	23.0 (167)	10.2 (74)	35.4 (257)*
High	55.7 (568)	21.1 (215)	10.6 (108)	30.7 (278)
*Sex:*				
Boys	52.8 (458)	22.8 (198)	11.9 (103)*	36.4 (315)**
Girls	55.7 (489)	21.0 (184)	9.0 (79)	29.0 (255)
*Age:*				
10 - 14 years	56.3 (667)*	22.0 (261)	11.3 (134)!	31.4 (372)
15 - 19 years	49.9 (280)	21.6 (121)	8.6 (48)	35.3 (198)
*Residence:*				
Urban	67.5 (565)**	17.6 (147)	13.5 (113)**	27.5 (230)
Rural	42.1 (382)	25.9 (235)**	7.6 (69)	37.4 (840)**

**P < 0.001;*p < 0.05 [! Fisher's exact test p < 0.05]

### Socio- behavioral distribution of caries experience, treatment need and self reported oral health

All socio-demographic- and oral health behavioral variables that were statistically significantly associated with oral health outcomes in unadjusted analyses (Table 3), were analyzed using stepwise, logistic regression models with DMT>0, moderate to high need for dental treatment and poor self reported oral health as dependent variables. Table 4 depicts adjusted ORs for DMT>0, moderate to high treatment need and self reported oral health by socio-demographics and oral health behaviors. Place of residence, gender and age were entered in the first step, providing a model fit of Nagelkerke's R^2 ^= 0.011, Model Chi- Square 12,555, df = 3 p < 0.001, with all socio-demographic variables statistically significantly associated with DMT. Entering self reported dental attendance in the second step improved the fit of the model to Nagelkerke's R^2 ^= 0.052, Model chi square = 57,887, df = 4, p < 0.0001. In the final second step, place of residence, gender, age and dental attendance were all statistically significantly associated with DMT (model 1). In the final second step of model 2, wealth index, gender and dental attendance associated statistically significantly with moderate to high treatment need. Finally in the second step of model 3, mother's education, place of residence, sex, use of toothpaste and sugar intake were the most important predictors of poor self reported oral health. Although socio-demographic disparities persisted after adjusting for oral health related behaviors, the effect of wealth index on dental treatment need was attenuated after adjusting for dental attendance.

**Table 3:** Distribution of oral health outcomes by socio-demographics and oral health behavioral characteristics.

Variable	DMT> 0	Moderate/high need for treatment	Poor self reported oral health
*Wealth index:*	% (n)	% (n)	% (n)
1st poorest quartile	18.2 (84)	9.1 (42)	43.3 (204)
2nd poor quartile	20.4 (159)	11.5 (90)	44.6 (348)
3rd poor quartile	29.0 (20)	20.3 (14)*	59.4 (41)
4th least poor quartile	18.4(80)	9.4 (41)	45.5 (198)
*Mother's education:*			
Low	20.1 (157)	10.6 (83)	49.0 (382)*
High	19.3 (186)	10.8 (104)	42.0 (409)
*Father's education:*			
Low	20.1 (146)	10.9 (79)	46.0 (334)
High	19.3 (197)	10.6 (108)	44.8 (457)
*Sex:*			
Boys	17.5 (152)	9.2 (80)	48.4 (420)*
Girls	21.8 (191)*	12.2 (107)*	42.3 (371)
*Age:*			
10 - 14 years	18.3 (217)	9.6 (114)	46.2 (547)
15 - 19 years	22.5 (126)*	13.0 (73)*	43.5 (244)
*Residence:*			
Urban	17.9 (150)	9.7 (81)	48.5 (406)**
Rural	21.3 (193)*	11.7 (106)	42.4 (385)
*Sugar consumption:*			
Low intake	19.4 (155)	11.3 (90)	42.2 (337)
High intake	19.9 (188)	10.2 (97)	47.9 (454)*
*Tooth brushing:*			
Never or seldom	19.1(73)	9.4 (36)	44.5 (170)
Once a day	19.3 (152)	11.2 (88)	48.4 (382)
More than once a day	20.6 (118)	11.0 (63)	41.6 (239)*
*Dental attendance:*			
No	17.5 (273)	9.1 (142)	44.8 (700)
Yes	38.5 (70)**	24.7 (45)**	50.0 (91)
*Use of F- toothpaste*			
No	19.3 (110)	9.5 (54)	51.6 (294)**
Yes	19.8 (233)	11.3 (133)	42.3 (497)

**p < 0.001, *p < 0.05

**Table 4:** Odds ratios (ORs) and 95% Confidence interval (CI) for caries experience (DMT>0), moderate to high need for treatment and poor oral health perceptions according to socio-demographic characteristics and oral health behaviors.

	Step 1: socio-demographics	Step 2: oral health behaviors
	OR (95% CI)	OR (95% CI)
***Model 1: DMT>0***		
Residence (rural versus urban)	1.2 (1.0 - 1.5)	1.3 (1.1 - 1.6)
Sex (girls versus boys)	1.4 (1.3 - 1.6)	1.5 (1.3 - 1.7)
Age (15-19 versus 10-14 years)	1.3 (1.0 - 1.7)	1.3 (1.1 - 1.4)
R squared	0.011	
Dental attendance (yes versus no)		3.7 (3.1 - 4.5)
R^2^		0.052
***Model 2: Moderate/high treatment need***		
Wealth: (2^nd ^poor quartile versus 1^st ^poorest quartile)	1.3 (0.9 - 1.9)	1.3 (0.9 - 1.9)
Wealth: (3^rd ^poor quartile versus 1^st ^poorest quartile)	2.6 (1.3 - 5.1)	2.2 (1.1 - 4.4)
Wealth: (4^th ^least poor quartile versus 1^st ^poorest quartile)	1.1 (0.7 - 1.8)	1.0 (0.6 - 1.6)
Sex (girls versus boys)	1.4 (1.1 - 1.9)	1.5 (1.1 - 2.1)
Age (15-19 versus 10-14 years)	1.5 (1.1 - 2.1)	1.6 (1.1 - 2.2)
R squared	0.021	
Dental attendance (yes versus no)		3.5 (2.4 - 5.1)
R^2^		0.044
***Model 3: Poor oral health perception***		
Mother's education (High versus Low)	0.9 (0.7 - 1.1)	0.9 (0.7 - 1.1)
Residence (rural versus urban)	0.7 (0.6 - 0.8)	0.7 (0.6 - 0.9)
Sex (girls versus boys)	0.8 (0.6 - 0.9)	0.8 (0.7 - 0.9)
R^2^	0.017	
Toothpaste use (yes versus no)		0.6 (0.5 - 0.8)
Sugar intake (high versus low)		1.2 (0.9 - 1.4)
Tooth brushing: Once a day versus never/seldom		0.9 (0.8 - 1.0)
Tooth brushing: >once a day versus never/seldom		0.9 (0.8 - 1.0)
R^2^		0.037

## Discussion

It is important to explore whether social disparities in adults' oral health present also in adolescents and whether the effects of the material distribution are mediated differently in adolescence and adulthood. This study revealed that a social gradient was present with respect to dental caries, treatment need, and reported oral health status as well as sugar intake, tooth brushing, use of fluoridated toothpaste and dental attendance patterns among adolescents in Kilwa, a generally deprived district with limited access to oral health care services. Differences across educational level, household wealth and place of residence groups were statistically significant for most oral health outcomes and oral health behaviors investigated, both in unadjusted and adjusted analyses. The results confirm previous findings in adolescent-and adult populations, globally [1,10,12,26]. Adolescents belonging to the less poor households presented with treatment need and dental attendance more frequently than their counterparts in the poorest households. According to Grytten and Holst [27], several US studies reported a positive association between income and demand for dental care, particularly when treatment need was high. Although dental attendance, oral hygiene behavior and sugar intake varied systematically with oral health outcomes (Table 3), social disparities in caries experience and self reported oral health were not attenuated whilst adjusting for those lifestyle patterns (Table 4). In spite of some attenuation of the relationship between household wealth and treatment need after controlling for dental attendance (OR reduced from 2.6 to 2.2), a direct relationship persisted that was unexplained by adolescent's dental attendance profile. Thus, the present results accord at best partly with findings from industrialized countries, suggesting that unequal access to dental care explains socio-economic disparities in adults' oral health [7,15,28]. In contrast to the experience from US, suggesting that children with untreated caries are less likely to obtain regular dental care, the present study indicate that Tanzanian adolescents who had attended a dentists were those with the most severe caries in terms of moderate to high treatment need [29]. This is a common finding in developing countries [30,31] and might reflect that dental attendance follows from a high treatment need rather than being an unexpected outcome of dental care. It might also reflect delayed treatment demand and limited access to dental care (only one assistant dental officer in Kilwa).

A low caries prevalence of about 20% presented among a minority of the participants is consistent with the caries trends of younger age groups in Tanzania [18,32,33]. In accordance with a suggested positive relationship between dental caries and the level of social development, previous studies have provided evidence of a higher caries prevalence in urban than in rural societies [10,12,34]. In contrast, the present findings accord to some extent with those observed among Brazilian schoolchildren, where living in a rural area almost doubled the odds of having dental caries [35]. Rural participants showed the highest prevalence of dental caries, but they were less dissatisfied with their oral health and visited a dentist less frequently than their urban counterparts. This is consistent with previous studies of schoolchildren in sub Saharan Africa [12,36]. Affordability, accessibility and structural barriers reflected by place of residence as a more area based measure of deprivation, have previously been reported to be important reasons for rural dwellers' non-use of dental services in Tanzania [36]. Nevertheless, independent of socio-economic position, students with caries experience and moderate to high need for dental treatment were the most frequent dental attendees suggesting a demand for dental care among young people in Kilwa.

The Lorenz curves presented in this study were based on the participating rural and urban population groups. Each group showed almost similar skewness dominance. Previously polarization mostly has been reported from western industrial populations. Macek *et al*. [37] described highly skewed caries data from young children, while the caries data from adolescents were more dispersed. However, more recent data of 15-yr-old Danish children collected from the Danish National Board of Health in 2006 [38], were more comparable to the present findings. Seventy-five percent of the total number of DMF-surfaces was found in 13% of Danish 15-yr-old children with the highest DMFS. It is interesting that the caries distribution among adolescents in a poor Tanzanian district is comparable to western industrial populations.

Socio-economic status of parents as assessed in terms of income, education and occupation, might be difficult to assess because of unawareness and unwillingness to reveal such information on the part of the adolescents, resulting in high rates of non responses particularly among lower socio-economic status groups [39]. The present study assessed socioeconomic status using a wealth index based on a weighted sum of self-reported household assets and the more conventional measure of parental education [1,21]. The wealth index showed good discriminate power against moderate to high need for treatment and oral health related behaviors even in the small, social homogeneous and generally deprived district of Kilwa. It bears similarity to the family affluence scale, FAS, developed as a supplementary measure of socioeconomic status for adolescents by the WHO Collaborative cross national study of Health Behavior in School aged Children [26]. Both the weighted wealth index and the FAS contain items reflecting family expenditure and consumption that are relevant to the family circumstances. In addition, this study used a surrogate area based social indicator of place of residence, as suggested by Locker [1].

The present study contributes to the knowledge of adolescents' oral health situation in deprived areas of Tanzania. However, the present results should be interpreted in the light of limitations that include a cross- sectional design and use of self-reported measures. Some schools in the selected wards were not accessible due to natural calamities in the area at the time of data collection and the number of enrolled standard six adolescents and attendance rates in rural schools were particularly low. Nevertheless, use of an unequal sampling fraction in urban and rural parts of Kilwa was compensated by providing weighted estimates for all oral health outcomes and oral health behaviors investigated. Since the present data rely on self reporting, they might have been biased by under-and over reporting due to socially desirable responses and poor recall effect. However, the core questions utilized in this study have shown a good validity and reliability in previous studies focusing children, adolescents and adults in sub Saharan Africa. The self-reported use of fluoridated toothpaste is questionable as we did not validate whether the toothpaste used contained the recommended free fluoride ions concentration. As it has been reported that toothpaste manufactured and sold in Tanzania has free fluoride concentrations below the recommended levels for prevention of caries [40]. Given that the data collection was conducted in a relatively short period of time, the presence of any temporal changes could hardly have confused the data.

## Conclusions

Substantial proportions of adolescents reported oral health detrimental behaviors indicating that there is room for improving oral self care, diet and access to and utilization of dental services among Tanzanian adolescents. In spite of demonstrating strong social disparities across oral health and oral health behaviors, sugar intake, oral hygiene and dental attendance patterns did not explain the socio-economic gradient in oral health status. Developing policies and programs targeting both social structural and individual behavioral determinants of oral health should be an urgent public oral health strategy in Tanzania.

## Competing interests

The authors declare that they have no competing interests.

## Authors' contributions

KOM: Principal investigator, conceived of the study, designed the study, collected data, statistical analysis and manuscript writing. ANA: Main supervisor, designed study, statistical analysis and manuscript writing. JRM: Participated in design of study. MSS: Have commented on the paper and provided valuable guidance for manuscript write up. All authors read and approved the final manuscript.

## Pre-publication history

The pre-publication history for this paper can be accessed here:

http://www.biomedcentral.com/1472-6831/10/7/prepub
